# A Brazilian cohort of pregnant women with overt diabetes: analyses of risk factors using a machine learning technique

**DOI:** 10.20945/2359-3997000000628

**Published:** 2023-05-29

**Authors:** Angela J. Reichelt, Maria Amélia A. de Campos, Vânia N. Hirakata, Vanessa K. Genro, Maria Lúcia R. Oppermann

**Affiliations:** 1 Hospital de Clínicas de Porto Alegre Porto Alegre RS Brasil Hospital de Clínicas de Porto Alegre, Serviço de Endocrinologia e Metabologia, Porto Alegre, RS, Brasil; Universidade Federal do Rio Grande do Sul, Programa de Pós-graduação em Ciências Médicas: Endocrinologia, Porto Alegre, RS, Brasil; 2 Hospital Nossa Senhora da Conceição Porto Alegre RS Brasil Hospital Nossa Senhora da Conceição, Serviço de Endocrinologia, Porto Alegre, RS, Brasil; 3 Hospital de Clínicas de Porto Alegre Unidade de Bioestatística e Análise de Dados Porto Alegre RS Brasil Hospital de Clínicas de Porto Alegre, Unidade de Bioestatística e Análise de Dados, Porto Alegre, RS, Brasil; 4 Hospital de Clínicas de Porto Alegre Porto Alegre RS Brasil Hospital de Clínicas de Porto Alegre, Serviço de Ginecologia e Obstetrícia, Porto Alegre, RS, Brasil; 5 Universidade Federal do Rio Grande do Sul Faculdade de Medicina Porto Alegre RS Brasil Universidade Federal do Rio Grande do Sul, Faculdade de Medicina, Porto Alegre, RS, Brasil

**Keywords:** Overt diabetes, pregestational diabetes, risk factors, machine learning technique

## Abstract

**Objective:**

Pregnancy complicated by type 2 diabetes is rising, while data on type 2 diabetes first diagnosed in pregnancy (overt diabetes) are scarce. We aimed to describe the frequency and characteristics of pregnant women with overt diabetes, compare them to those with known pregestational diabetes, and evaluate the potential predictors for the diagnosis of overt diabetes.

**Subjects and methods:**

A retrospective cohort study including all pregnant women with type 2 diabetes evaluated in two public hospitals in Porto Alegre, Brazil, from May 20, 2005, to June 30, 2021. Classic and obstetric factors associated with type 2 diabetes risk were compared between the two groups, using machine learning techniques and multivariable analysis with Poisson regression.

**Results:**

Overt diabetes occurred in 33% (95% confidence interval: 29%-37%) of 646 women. Characteristics of women with known or unknown type 2 diabetes were similar; excessive weight was the most common risk factor, affecting ~90% of women. Age >30 years and positive family history of diabetes were inversely related to a diagnosis of overt diabetes, while previous delivery of a macrosomic baby behaved as a risk factor in younger multiparous women; previous gestational diabetes and chronic hypertension were not relevant risk factors.

**Conclusion:**

Characteristics of women with overt diabetes are similar to those of women with pregestational diabetes. Classic risk factors for diabetes not included in current questionnaires can help identify women at risk of type 2 diabetes before they become pregnant.

## INTRODUCTION

Pregnancy associated with type 2 diabetes is rising, following the burden of excessive weight in women of childbearing age (1). The glycemic status of a woman is crucial to reduce unfavorable outcomes for mothers and fetuses. Overt diabetes, hyperglycemia reaching non-pregnant criteria for diabetes and first diagnosed in pregnancy (2), can be as hazardous as the already-known type 2 diabetes (1). Undiagnosed diabetes is not uncommon in adults (3), but few data exist regarding this condition in pregnancy. In a Canadian study, 2.6% of 68 163 women evaluated up to one year after gestational diabetes presented a diagnosis of type 2 diabetes, pointing to a likely diagnosis of overt diabetes in pregnancy (4); in a Brazilian cohort, 48 of the 224 pregnant women with hyperglycemia (21.4%) fulfilled diagnostic criteria for overt diabetes (5). Larger studies on type 2 diabetes characteristics and outcomes in pregnancy excluded women with overt diabetes (6,7).

Several predictors have been proposed to assess the risk of diabetes in adults (8-10). Many questionnaires set 40 (9) or 45 years (8,10) as the lowest age, limiting its adoption to most women of childbearing age. Moreover, except for previous gestational diabetes, no other questions on obstetric antecedents appear in those questionnaires.

Therefore, we aimed to: describe the frequency of diabetes with the first diagnosis in pregnancy, i. e. overt diabetes; compare characteristics of women with overt diabetes to those with known pregestational type 2 diabetes; and evaluate factors that could identify, before pregnancy, women at risk of presenting a diagnosis of overt diabetes.

## SUBJECTS AND METHODS

In this retrospective cohort study, we included all pregnant women receiving high-risk antenatal care in the two major public hospitals (*Hospital de Clínicas de Porto Alegre* (11) and *Hospital Nossa Senhora da Conceição* (12) in Porto Alegre, Brazil, from May 20, 2005, to June 30, 2021.

The study protocol was approved on July 28, 2016 (number 16-0331) and registered in Plataforma Brasil, CAAE 57365016.3.0000.5327; all authors signed a data use agreement form to ensure the privacy of data collected from medical registries.

We included all women with known pregestational type 2 diabetes; and all those fulfilling the 2013 World Health Organization criteria for overt diabetes (13) and/or glycated hemoglobin (HbA1c) ≥ 6.5% (9). We did not exclude women with twin pregnancies. We included data from the first pregnancy in women who became pregnant more than once during the study span. We excluded women with type 1 diabetes, gestational diabetes, or an unclear diagnosis of hyperglycemia. A multi-professional team provided care at both hospitals.

We followed the STROBE (Strengthening the Reporting of Observational Studies in Epidemiology) statement to write the manuscript (14).

Duration of diabetes and pre-pregnancy weight were self-informed. We categorized skin color as white or non-white; and education, as more than 11 years or 11 years or less of formal education. The presence of diabetes complications, smoking, family history of diabetes or chronic hypertension, personal history of hypertension, previous gestational diabetes, or macrosomia (birth weight ≥ 4,000 g) were considered positive when reported in the hospital charts. The same was applied to a family history of diabetes or hypertension in relatives of the first or second degree. Absent information on these variables was labeled as negative.

Height and weight were measured at the first prenatal appointment. Pregestational BMI was calculated as the informed weight in kilograms divided by the square of height, in meters, and women were classified as having normal BMI, overweight, or obesity (15).

HbA1c was measured at booking and labeled as initial HbA1c, regardless of gestational age. Assays were conducted with high-performance liquid chromatography (Variant II Turbo HbA1c; BioRad, Hercules, CA, USA) in line with the National Glycohemoglobin Standardization Program guidelines (http://www.ngsp.org/index.asp).

We calculated the frequency of women with overt diabetes and compared their baseline characteristics to those with known pregestational type 2 diabetes using univariable analysis.

We applied two approaches to evaluate possible risk factors with overt diabetes as the dependent variable. The machine learning technique was used as an exploratory tool, while multivariable analyses estimated relative risks for each factor.

The machine learning technique analyses included all risk factors in the model; the program layered them. Data were tested using either continuous or categorized variables to generate the models. Cross-validation was used as the sampling method. We chose the number of folds the dataset should be split based on the best resulting area under the curve (AUC). Each fold represents the number of splits the dataset was divided, to train and test the model. In cross-validation, the training and testing subsets are trained and tested according to the selected number of folds. The decision tree model was chosen as the algorithm. Besides AUC, precision (positive predictive value) and recall (sensitivity) were also retrieved.

After this preliminary analysis, we ran models with the ADA's “Are you at risk for type 2 diabetes” questionnaire (9) as the matrix. We entered age, continuous or dichotomized; previous gestational diabetes (no/yes); family history of diabetes (no/yes); personal history of chronic hypertension (no/yes); and pregestational BMI, continuous or categorized as normal, overweight, or obesity. Two items of the questionnaire were excluded: question 2 (gender) and question 6 (physical activity, information not available in the dataset). Obstetric variables included the number of deliveries, continuous or dichotomized; previous miscarriage (no/yes); and previous macrosomia (no/yes). Women with overt diabetes were compared to those with pregestational type 2 diabetes by estimating relative risks for main risk factors.

Three models were chosen. The first included all risk factors; then, we included only variables of the ADA's risk questionnaire; and finally, we evaluated a combination of ADA's risk score plus risk factors related to pregnancy: number of deliveries dichotomized as ≥ 2 and history of previous macrosomia.

Statistical analyses were performed with SPSS version 18.0 (SPSS, Chicago, IL, USA). Results were expressed as mean ± standard deviation (SD) or median (interquartile range, IQR) according to a normal distribution as determined by Shapiro-Wilk test, or number (percentage). The Student *t*-test, the chi-square test (coupled with the *Z* test for comparison of proportions, with Bonferroni correction when appropriate), and the Mann-Whitney *U* test were used to compare baseline characteristics of women with overt diabetes to those with pregestational diabetes.

We used the Orange Workflow version 3.30.2 for machine learning analyses; relative risk (RR) with 95% CI was estimated using Poisson regression and was performed with SPSS.

## RESULTS

We enrolled 648 women; we excluded two due to missing information on diabetes duration. Data on 127 women with type 2 diabetes from HCPA have been previously described (16).

Overt diabetes was diagnosed in 212 women (33.0%, 95%CI 29.0-37.0%); 116 (54.7%) were in the first trimester, 64 (30.2%), in the second and 32 (15.1%), in the third. Diagnostic tools are in Table 1.

**Table 1:** Diagnosis of overt diabetes in 212 pregnant women

Tool	n (%)
Only FPG	34 (16.0)
Only HbA1c	19 (9.0)
Only 2-hour glucose (OGTT)	30 (14.2)
2-hour (OGTT) + HbA1c	7 (3.3)
FPG + 2-hour glucose (OGTT)	14 (6.6)
FPG + HbA1c	72 (34.0)
All (OGTT + HbA1c)	36 (17.0)

FPG: fasting plasma glucose; HbA1c: glycated hemoglobin; OGTT: oral glucose tolerance test.

The baseline characteristics of women are in Table 2. The median number of deliveries was 1.0 [IQR 1.0-2.0]. One hundred and one women (47.6%) with overt diabetes and 206 (47.5%) women with pregestational diabetes had > two deliveries (p = 1.000).

**Table 2:** Characteristics of pregnancies in women with type 2 diabetes according to the moment of diagnosis

Characteristic		Diabetes			p^a^
		All	Overt	Pregestational	
		n = 646	n = 212 (33.0)	n = 434 (67.0)	
Center					0.584
	HCPA	304 (47.1)	96 (45.3)	208 (47.9)	
	HNSC	342 (52.9)	116 (54.7)	226 (52.1)	
Age (years)		33 (5.9)	32 (6.1)	33 (5.7)	0.001
White skin color		449 (69.5)	150 (70.8)	299 (68.9)	0.696
Schooling level (≤11 years)		612 (94.7)	203 (95.8)	409 (94.2)	0.534
Smoking		55 (8.5)	30 (6.9)	25 (11.8)	0.053
Duration of diabetes (years)		-----	-----	4.0 [2.0-7.0]	-----
				433	
Gestational age at diagnosis (weeks)		-----	12.3 [8.2-19.0]	-----	-----
Diabetes complications		41 (6.3)	2 (0.9)	39 (9.0)	<0.001
Chronic hypertension		148 (22.9)	38 (17.9)	110 (25.3)	0.045
Family history of diabetes		431 (66.7)	131 (61.8)	300 (69.1)	0.077
Family history of hypertension		324 (50.2)	102 (48.1)	222 (51.2)	0.521
Number of pregnancies		3.0 (1.7)	3 (1.6)	3 (1.8)	0.216
First pregnancy		116 (18.0)	39 (18.4)	77 (17.7)	0.925
Previous miscarriage		186 (28.8)	55 (25.9)	131 (30.2)	0.305
Previous gestational diabetes		204 (31.6)	66 (31.1)	138 (31.8)	0.936
Previous macrosomia		129 (20.0)	51 (24.1)	78 (18.0)	0.087
Pregestational BMI (kg/m^2^)		34.3 (7.6)	34.7 (8.2)	34.2 (7.4)	0.363
		623	205	418	
BMI categories					0.836
Normal		57 (9.1)	19 (9.3)	38 (9.1)	
	overweight	124 (19.9)	38 (18.5)	86 (20.6)	
	obesity	442 (70.9)	148 (72.2)	294 (70.3)	
		623	205	418	
Gestational age at booking (weeks)		19.2 [13.3-27.0]	24.4 [18.0-31.1]	17.4 [12.2-24.0]	<0.001
HbA1c at booking (%)		7.3 (1.6)	7.0 (1.3)	7.4 (1.7)	0.002
		638	211	427	

HbA1c: glycated hemoglobin; HCPA: Hospital de Clínicas de Porto Alegre; HNSC: Hospital Nossa Senhora da Conceição.

Data are presented as mean (standard deviation) or number (%) or median [interquartile range].

a p values were analysed by χ2 test for categorical variables; Student t test for continuous variables with normal distribution; Mann-Whitney for continuous variables with non-normal distribution.

**Figure 1:**
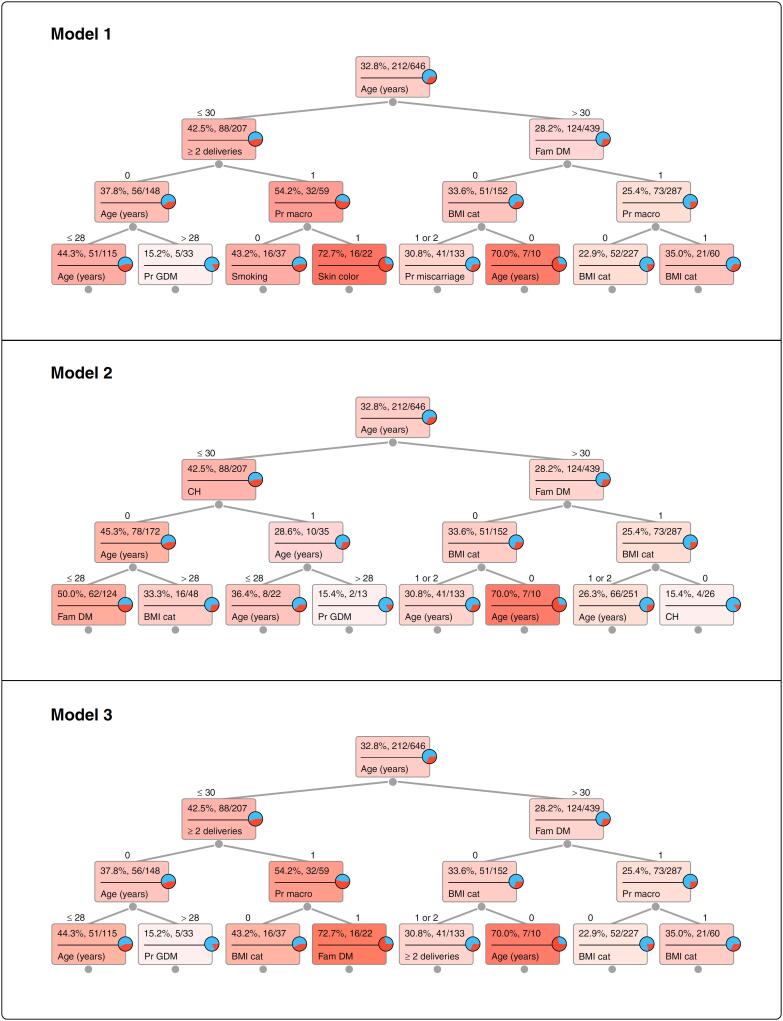
Machine learning analysis of risk factors for overt diabetes. BMI cat: body mass index categories (normal BMI, overweight, obesity); CH: chronic hypertension; Fam DM: family history of diabetes; Pr GDM: previous gestational diabetes; Prv macro: previous macrosomia. 0 = no; 1 = yes; for BMI cat: 0 = normal; 1 = overweight; 2 = obesity. Each model corresponds to a model with the same number in Table 3.

Results of the machine learning algorithm are displayed in Table 3 and illustrated in the Figure 1. AUC was under 0.6 in all models; we chose the three models with the higher AUCs. Model 1 included, besides the classical risk factors of ADA's questionnaire, demographic characteristics plus previous macrosomia and ≥ 2 deliveries, leading to an AUC of 0.594. Maternal age was the first factor to be dichotomized: into ≤ 30 years and > 30 years: 124 (58.5%) women were > 30 years in the group of overt diabetes and 315 (72.6%) in the pregestational type 2 diabetes group. This cutoff of age was used for all other analyses.

**Table 3:** Risk factors for overt diabetes: matrix and results using learning machine technique

Model^a^	Age	CH	Family DM	≥2 deliveries	Pr GDM	Pr macro	BMI cat	AUC	Precision	Recall	Folds
1	X	X	X	x	X	X	x	0.594	0.621	0.667	2
2	X	X	X		X		x	0.581	0.615	0.656	2
3	x	X	X	x	X	X	x	0.576	0.622	0.658	2

a Model AUC: area under the receiver-operating curve; BMI cat: body mass index categories (normal BMI, overweight, obesity); CH: previous chronic hypertension; DM: diabetes; precision: positive predictive value; Pr GDM: previous gestational diabetes; Prv macro: previous macrosomia; recall: sensitivity.

Model 1 includes all risk factors (age - continuous, skin color, schooling, smoking, family DM, CH, ≥2 deliveries, previous miscarriage, Pr GDM, Pr macrosomia, BMI categories).

Model 2 includes the five items of ADA's risk questionnaire (age - continuous, CH, family DM, Pr GDM, BMI categories).

Model 3 includes the five items of ADA's risk questionnaire (age - continuous, CH, family DM, Pr GDM, BMI categories) + ≥2 deliveries + previous macrosomia.

In Model 1, in women ≤ 30 years, the number of deliveries appeared at the second level, followed by previous macrosomia in those with ≥ 2 deliveries. A family history of diabetes appeared at the second level in women > 30 years, followed by BMI category in those without a family history of diabetes, and by previous macrosomia in those with a family history of diabetes. Demographic characteristics appeared at the fourth level.

Model 2 comprised the five items of ADA's questionnaire; AUC was 0.581. In this model, chronic hypertension was the second risk factor in women ≤ 30 years, while in those > 30 years, a history of family diabetes was the second.

In Model 3, we added previous macrosomia and ≥ 2 deliveries to Model 2. AUC was 0.576, and in women ≤ 30 years, the number of deliveries followed; previous macrosomia appeared at the third level in those with ≥ 2 deliveries. Family history of diabetes remained at the second level in women with age > 30 years, and previous macrosomia appeared at the third level in those with a family history of diabetes, while in those without, BMI was at the third level.

The results of the multivariable analysis are in Table 4. An age cutoff of > 30 years conferred a 33% lower risk and family history of diabetes, a 20% lower risk of overt diabetes, while a history of previous macrosomia enhanced the risk by 32%. Chronic hypertension was not significant after adjustments.

**Table 4:** Maternal characteristics as risk factors for overt diabetes

		Model 1	p	Model 2	p	Model 3	p
Age > 30 years		0.67 (0.53-0.85)	0.001	0.67 (0.53-0.83)	<0.001	0.67 (0.53-0.83)	<0.001
Non-white skin color		0.94 (0.74-1.20)	0.614				
Chronic hypertension		0.80 (0.59-1.08)	0.142	0.77 (0.57-1.05)	0.094	0.79 (0.58-1.07)	0.124
Family history of DM		0.80 (0.63-1.00)	0.050	0.80 (0.64-1.00)	0.054	0.80 (0.63-0.99)	0.047
Previous GDM		0.97 (0.74-1.25)	0.791	1.04 (0.82-1.32)	0.746	0.96 (0.75-1.24)	0.768
Previous macrosomia		1.32 (1.01-1.75)	0.049			1.32 (1.01-1.71)	0.040
BMI category							
	overweight	1.00 (0.64-1.54)	0.986	0.97 (0.62-1.51)	0.884	0.98 (0.63-1.52)	0.936
	obesity	1.03 (0.71-1.51)	0.874	1.04 (0.71-1.53)	0.834	1.03 (0.70-1.50)	0.900
≥2 deliveries		1.00 (0.92-1.09)	0.934				
Previous miscarriage		0.91 (0.71-1.18)	0.484				

DM: diabetes mellitus; GDM: gestational diabetes; BMI: body mass index.

Data presented as adjusted relative risk and 95% CI.

Model 1 – includes all variables: age, skin color, schooling, smoking, family DM, chronic hypertension, ≥2 deliveries, previous miscarriage, previous GDM, previous macrosomia, BMI categories (normal, overweight, obesity).

Model 2 – based on ADA's questionnaire of risk; age, BMI categories (normal, overweight, obesity).

Model 3 – based on ADA's questionnaire of risk + previous macrosomia; age, BMI categories (normal, overweight, obesity).

## DISCUSSION

Diabetes was unveiled for the first time in one-third of this cohort of women with diabetes. Baseline characteristics of women with known or unknown pregestational diabetes were similar; ~6.0% of women presented chronic complications of the disease, mainly those with pregestational diabetes. An age of > 30 years and a positive family history of diabetes were inversely associated with the diagnosis of overt diabetes; in younger women with two or more deliveries, and in those > 30 years with a family history of diabetes, previous macrosomia was a predictor of overt diabetes. Prior GDM history and chronic hypertension did not discriminate groups.

In Brazil, diabetes occurs in 0.9% of women aged 18-24 years and in 5.7% of those aged 35-44 years (17). In the ELSA-Brasil study, 5.1% of women aged 35-44 years were diagnosed with diabetes; 56.9% were unaware of having hyperglycemia (18), in contrast to lower figures found in the American population (3). One-third of the participants were unaware of the diagnosis in our study, mirroring numbers reported for non-pregnant women. Women with overt diabetes arrived later to the specialized prenatal care as expected, adding to the risk of hyperglycemia-related fetal malformations. Why did we not diagnose them before? A delayed diagnosis may be explained by the low socioeconomic and/or educational profiles in this sample, as illustrated by the high frequency of low schooling and pregnancy planning. Non-planned pregnancies are not exclusive to women with diabetes; in a cohort of Southern Brazilian women, 52.2% said their pregnancies were unplanned (19), mainly in the lower socioeconomic stratum. Could diabetes have been diagnosed before pregnancy? We believe yes if some risk factors had been sought.

Age is a relevant risk factor for type 2 diabetes and the first query in most questionnaires (8-10). Women presenting overt diabetes were only one year younger than those with pregestational diabetes here, a difference that does not explain why these women were not diagnosed before pregnancy. Current guidelines recommend that women < 35 years should only be screened for diabetes in the presence of excessive weight; or when pregnant. Age was the first risk factor to be dichotomized by the machine learning technique in our study; it was an independent risk factor in most multivariable analyses.

Excessive weight is probably the most relevant risk factor related to diabetes, both running in parallel in several world regions (20). Overweight and obesity rates are rapidly growing among women of childbearing age. A Brazilian survey revealed that obesity was present in 11.2% of women aged 18 to 24 years, and in ~26.0% of those aged 35 to 44 years, overweight ranged from 31.7% to 61.9% (17). More than 70% of women presented with obesity here. BMI is part of all risk scores for diabetes screening in women of childbearing age, either in those to detect diabetes by universal screening (21) or in those to predict the risk of diabetes in women with prior gestational diabetes (22). The dyad excessive weight/lower age recently prompted the United States Task Force decision to lower the age for diabetes screening to 35 years (23); had this rule been applied here, one-third of the women with overt diabetes would have been screened earlier.

Gestational diabetes is a well-known risk factor for presenting type 2 diabetes in the future (24). Previous gestational diabetes history, although present in ~30% of women here, was not discriminative, contrasting to findings of others (24): in machine learning models, it would appear at third or fourth levels; and it was not significant in multivariable analyses. A positive history of gestational diabetes was a relevant factor in a Mexican risk score for incident diabetes screening in women of childbearing age (25).

A family history of diabetes points to an underlying genetic and/or environmental factor. A positive family history of diabetes was inversely associated with overt diabetes, in adjusted models, probably because having cases of diabetes in the family led women to seek earlier screening, and it only appeared at the second or third levels in the learning machine algorithms. Family history almost doubled the chance of undiagnosed diabetes in one study that compared people with and without diabetes (26).

Chronic hypertension was more common in women with pregestational diabetes, although they were neither older nor heavier. They presented diabetes for at least the previous four years, and diabetes complications were more frequent. Chronic hypertension was present in 17.6% of women aged 35 to 44 years in Brazil (17), similar to that found in younger women with overt diabetes, but lower than the frequency in women with pregestational diabetes. Hypertension was not relevant in adjusted models here, in opposition to the findings of others (26), which included only non-pregnant women and older participants; it was also not relevant in a cohort of Mexican women of childbearing age (25).

Delivery of macrosomic babies is linked to maternal weight and hyperglycemia (27) and was associated with an increased risk of future type 2 diabetes, irrespective of earlier gestational diabetes (28). The frequency of macrosomic babies born to women without type 2 diabetes was 5.9% in one study (29) and 7.8% in another (30). Macrosomia occurred in 11.5% of the women without previous gestational diabetes, and 38.2% of those with prior gestational diabetes, and was associated with an increased risk of overt diabetes.

The main message of this study is that it is not enough to measure glycemia or HbA1c in the first trimester of pregnancy. We dare to say that screening for type 2 diabetes has to begin before conception to ensure the benefits that the knowledge of having diabetes can provide to women of childbearing age (31). Chronic complications were probably unexpected due to the short length of diabetes in both groups; nevertheless, they occurred in ~6.0 % of women. Diabetes complications were reported in 0.7% of women with overt diabetes in another study (32), compared to 0.9% here, and in 3.2% of those with known pre-pregnancy type 2 diabetes (32), compared to 5.5% here.

Based on risk factors, women could be diagnosed before they become pregnant. Risk scores based on ADA's questionnaire and including a lower age stratum (30 years) plus the information on the delivery of a macrosomic baby might help to identify diabetes in childbearing-age women.

Our study has strengths: we evaluated a large group of women with hyperglycemia in pregnancy and compared them to those with known pregestational diabetes. Specific risk factors were found according to the age of women, despite the similarity of the groups. The results also suggest that we need to anticipate the screening for type 2 diabetes in women of childbearing age.

Limitations of the study must be cited. We included data retrieved from medical registries; we assumed risk factors not recorded in medical charts as absent, probably underestimating their actual frequency. Regarding lack of information on lifestyle, in a Brazilian survey, only ~35% of women of childbearing age declared they exercised regularly, and less than 40% ate fruits and vegetables five or more days a week (17). This way, we assumed that lifestyle information would not significantly impact our results. Low precision estimates, like the low AUCs found for the risk factors models, may reflect the similarity between the two groups. We could not re-evaluate women with overt diabetes after delivery to confirm the diagnosis of diabetes, nor could we diagnose potential cases of Maturity Onset Diabetes of Youth (MODY) among women classified as having type 2 diabetes due to technical limitations to carry out genetic tests as routine care. MODY accounts for only ~1% of pregnancy-associated diabetes (33), and we believe this limitation did not impact our results. Lastly, we did not test our risk score in pregnant women without diabetes; they would probably perform better had we included these women.

In conclusion, classic risk factors could identify women at risk of type 2 diabetes before they become pregnant. Setting a lower age cut point and including the previous delivery of a macrosomic baby in the current screening questionnaires could improve their performance in reproductive-aged women.
